# GJB2 mutations in Iranian Azeri population with autosomal recessive nonsyndromic hearing loss (ARNSHL): First report of c.238 C>A mutation in Iran

**DOI:** 10.1002/jcla.24024

**Published:** 2021-09-28

**Authors:** Ehsan Abbaspour Rodbaneh, Mohammad Panahi, Bahareh Rahimi, Haleh Mokabber, Reza Farajollahi, Behzad Davarnia

**Affiliations:** ^1^ Medical Genetics and Pathology Ardabil University of Medical Sciences Ardabil Iran; ^2^ Department of Medical Biotechnology Faculty of Advanced Medical Sciences Tabriz University of Medical Sciences Tabriz Iran; ^3^ Department of Medical Biotechnology Faculty of Allied Medical Sciences Iran University of Medical Sciences Tehran Iran; ^4^ Ardabil Welfare Organization Ardabil Iran

**Keywords:** Azeri population, gap junction protein beta 2, GJB2, hearing loss, Iran, nonsyndromic hearing loss

## Abstract

**Objective:**

Autosomal‐recessive nonsyndromic hearing loss (ARNSHL) is a heterogeneous genetic disorder. Mutations in the gap junction protein beta 2 (GJB2) gene, encoding connexin 26, are a significant cause of ARNSHL in different ethnic groups. This study aimed to identify the frequency and type of GJB2 mutations in the Iranian Azeri population.

**Methods:**

Fifty unrelated families presenting ARNSHL in Ardabil Province, the northwest of Iran, were studied to determine the frequency and type of GJB2 mutations leading to ARNSHL. ARMS‐PCR screened all DNA samples to detect c.35delG; p. Gly12Val mutation. In addition, normal samples for c.35delG; p. Gly12Val were analyzed by direct sequencing for other GJB2 mutations.

**Result:**

Of the fifty families, 13 (26%) showed a GJB2 gene mutation, with c.35delG; p. Gly12Val mutation was the most prevalent one that occurred in eight (61.5%) out of the 13 families. Of the families, two were homozygous for c.358‐360delGAC; p. Glu120del mutation, and one was homozygous for c.290dupA; p. Tyr97Ter and c.299–300delAT; p. His100Arg mutations. Also, we detected a novel mutation, c.238C>A; p. Gln80lys, in one of the families.

**Conclusion:**

Our findings are comparable to previous studies, indicating c.35d3lG; p. Gly12Val mutation in the GJB2 gene is the most common cause of GJB2‐related hearing loss in the Iranian Azeri population. Furthermore, our study highlights the significance of ARNSHL screening programs of live births based on local population data in Iran.

## Graphical abstract

The data from the 50 families in Ardebil Province with ARNSHL were analyzed, and GJB2 mutations were detected in 13 (26%) of the families. The blood sample was taken from the patients with their consent, and DNA extraction was carried out. Screening of 35delG was performed using ARMS‐PCR, and eight of the families were observed with 35delG in the homozygous form, accounting for about 61.5% of GJB2‐related hearing loss in the study. Afterward, sequencing of GJB2‐exon 2 was performed for the other families, and four different new mutations were identified. Moreover, c.358‐360delGAG was recognized in two of the families in the homozygous form. Also, three other mutations, including c.238C>A, c.299‐300delAT, and c.290DupA, were found in three different patients, of which c.238C>A was reported for the first time in Iran.

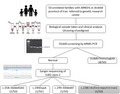

## 1 INTRODUCTION

Hearing loss (HL) is a common sensory disorder that affects millions of people worldwide, with an incidence of one in every 500 newborns (http://hearing.screening.nhs.uk/nationalprog). There are two different genetic forms of HS, including syndromic and nonsyndromic forms. There is a preponderance of nonsyndromic hearing loss (NSHL) in different populations, in which 70% represents an autosomal recessive pattern of inheritance.^1^ Autosomal recessive nonsyndromic hearing loss (ARNSHL) is highly heterogeneous, with about 100 mapped loci and over 70 causative genes (http://hereditaryhearingloss.org).^2^ In different populations, especially Asian and European descent, a mutation in GJB2 and GJB6 is a major cause of ARNSHL. GJB2 is located on the locus of DFNB1 (chromosome 13q12), which encodes the connexin 26 protein as a particular gap junction belonging to the families of intercellular channels for cell‐to‐cell interaction and small molecule distribution. GJB2 protein has been present in the human cochlea during the development of the embryo in the 22nd week, and it has an essential function in the homeostasis of the inner ear by recycling potassium ions.^3^

Meanwhile, several studies have shown in Iran that no mutation in the GJB6 gene is involved in congenital hearing loss. In other words, this probably suggests that mutations in GJB6 gene, especially deletions, clearly do not have a distinct effect on the etiology of ARNSHL among Iranian population. Therefore, the diagnostic value of genetic tests to detect mutations in the GJB6 gene as a first step would not be as valuable as determining mutations in the GJB2 gene. These findings demonstrated that GJB6 deletions were restricted to certain areas and populations, showing a funder effect regarding these mutations as well.^4^, ^5^, ^6^, ^7^, ^8^, ^9^

Different studies performed during the last decade indicate an ethnic bias in GJB2 mutation. For example, c.35delG; p. Gly12Val is highly the prevalent in Whites (about 85%), c.167delT; p.Leu56fs in Ashkenazi Jews, c.235delC; p.Leu79fs in Japanese, Chinese, and Korean people, c.109G>A; p.Val37Ile in Thai people, c.71G>A c.Trp24X in Indians, and c.427C > T; p.Arg143Trp in Ghanaian people.^10^ Since there are many different ethnic groups in Iran, it is necessary to produce ethnic‐organized data regarding GJB2 mutation. During the last decade, several studies have been carried out on different Iranian ethnic groups to identify the frequency and spectrum of GJB2 mutations. According to different scientific reports, GJB2‐related ARNSHL occurs in 16%–18% of Iranian populations, and c.35delG; p. Gly12Val is the most frequent mutation, leading to premature termination of the protein and GJB2‐related deafness.^11^, ^12^ This study aimed to determine the frequency and type of some GJB2 mutations (including the coding region of GJB2) in the Azeri people of Ardabil province in the northwest of Iran, a province with high consanguinity mating rate.^13^

## 2 MATERIALS AND METHOD

### 2.1 Subjects

In total, 50 non‐related families with ARNSHL from Ardabil were investigated. ARNSHL patients were selected by screening the pedigree of the families and their medical records, the patients’ audiologic testing, and the information of two or more patients in the families. There are no symptoms and signs other than HL. Patients were excluded if HL was due to environmental factors, such as intrauterine (eg, rubella virus) infections, ototoxic drugs (eg, aminoglycoside antibiotics), and noise exposure. Only ARNSHL individuals were recruited from families with segregated hearing impairment, including two or more patients. Before beginning the research, written informed consent was obtained from the patients and their families. Then, an analysis of DNA extraction and amplification was conducted.

### 2.2 Clinical evaluation of proband with new mutation c.238C>A; p. Gln80lys

The affected proband is the second offspring of the family in which the parents have a more distant relationship according to their relatives. He was 61 years old with ARNSHL, and his mother and father had passed away (Figure 3).

### 2.3 DNA extraction

About 6 ml of peripheral blood was obtained from the patients and their parents and siblings at any time possible and collected in EDTA‐containing tubes (0.5 M); subsequently, genomic DNA was extracted from peripheral leukocytes using the standard salting‐out method.^14^ Finally, the purity and integrity of extracted DNA samples were assessed with the Nano drop spectrophotometer (Thermo Fisher Scientific) and agarose gel electrophoresis, respectively.^15^

### 2.4 The genetic analysis of the gap junction protein beta 2 (GJB2)

Mutation in the GJB2 (exon2) coding region was amplified and screened using PCR and direct sequencing, respectively. At first, allele‐specific PCR amplification (ARMS PCR) was used with specific primers according to the Scott et al.’s procedure^16^ to identify c.35delG mutation in the GJB2 gene. Then, in a normal individual without c.35delG mutation, the entire exon2 of GJB2 was sequenced. For the amplification of exon2, the following primers were used: F1 (5'‐TGC TTG CTT ACC CAG ACT CAG‐3’) and R1 (5'‐GGT TGC CTC ATC CCT CTC AT‐3’) and F2 (5'‐ GTG GAC CTA CAC AAG CAG CA‐3’) and R2 (5'‐ TAA CAG CCT GGG GTC TCA GT‐3’). After amplification, BigDye Terminators (Applied Biosystems 3130 Genetic Analyzer) were applied to sequence PCR products.

### 2.5 Computational analysis

We used some bioinformatics tools, including SIFT, Mutation Taster, and ClinVar, to predict mutations’ effect on the connexin 26 protein structure. The pathogenicity of the detected variant was evaluated through SIFT, Mutation Taster, and the ClinVar webserver. According to the analysis of mutations using Mutation Taster, all the mutations were disease‐causing and based on ClinVar analysis, all of them were pathogenic. SIFT webserver analysis showed that some of the mutations were damaging (Table 1).

**TABLE 1:** GJB2 mutations and properties in the present study

Mutant variant	Zygosity	Effect on protein	No. Probands total sample	No. Proband with alteration in GJB2	Classification	Mutation Type	Mutation Taster	SIFT	ClinVar
c.35delG	HOM	p.Gly12Val	8/50=16%	8/13=61.5%	T	Deletion/Nonsense	Disease causing	Not available	Pathogenic
c.290DupA	p.Tyr97Ter	1/50=2%	1/13=7.6%	T	Duplication	Disease causing	Not available	Pathogenic
c.358‐360delGAG	p.Glu120del	2/50=4%	2/13=15.38%	NT	In‐frame deletion	Disease causing	Not available	Pathogenic
c.238C>A (First report in Iran)	p.Gln80Lys	1/50=2%	1/13=7.6%	NT	Missense	Disease causing	Damaging	Pathogenic
c.299‐300delAT	p.His100Arg	1/50=2%	1/13=7.6%	T	Deletion/Nonsense	Disease causing	Damaging	Pathogenic

## 3 RESULTS

**FIGURE 1:**
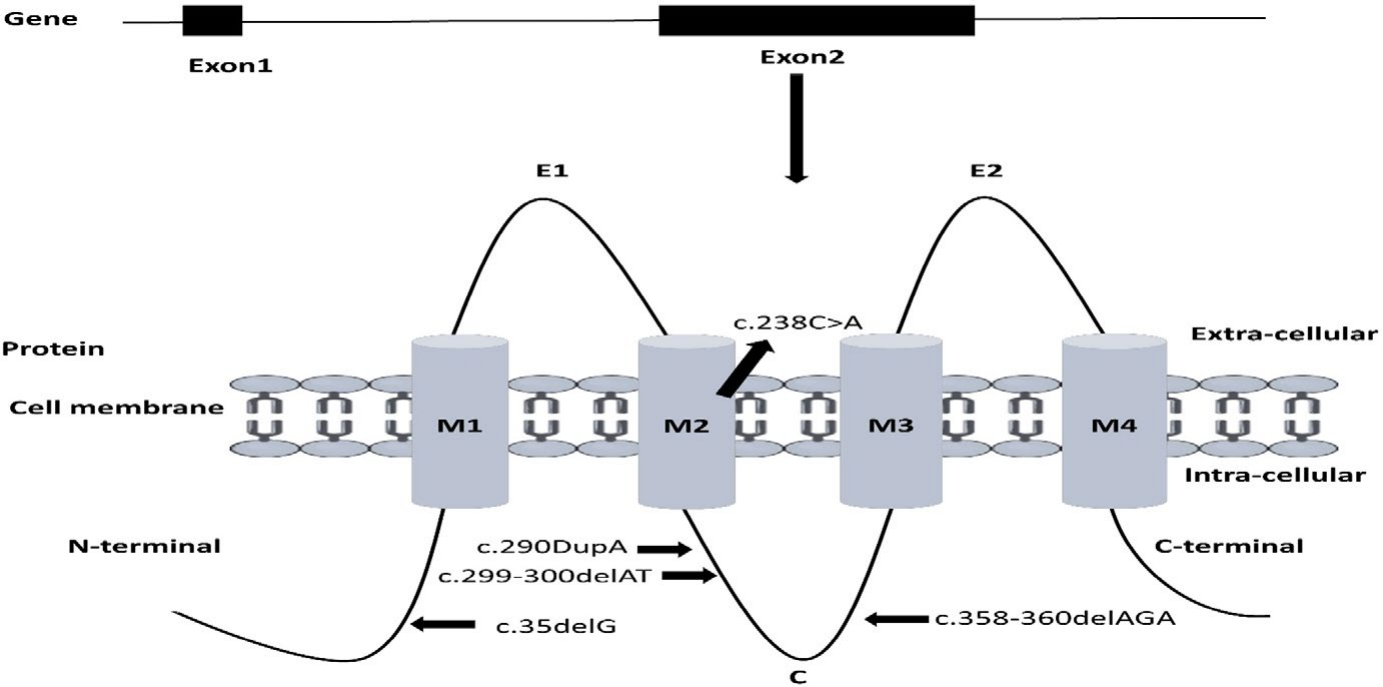
Effect and positions of different GJB2 mutations (exon2) on the protein domains

Data from 50 families with ARNSHL in Ardabil were analyzed, and GJB2 mutations were detected in 13 (26%) of the families (Table 1). The patients had an age range between 5–74 years. Of the 13 patients, 11 (84.6%) were males and three (23.07%) were females. In total, five homozygous variants were identified, with homozygous c.35delGl; p. Gly12Val being the most common mutation observed in eight (61.5%) of the families. Moreover, c.358‐360delGAG; p. Glu120del, which is an in‐frame deletion, was twice recognized in the homozygous form. Also, three other mutations, including c.238C>A; p. Gln80Lys, c.299‐300delAT; p. His100Arg, and c.290DupA; p. Tyr97Ter, were found in three of the patients, and c.238C>A among them was reported in Iran for the first time. This mutation was categorized as a pathogenic and missense mutation, leading to an amino‐acid exchange of glutamine 80 to lysine (Figure 1).

## 4 DISCUSSION

**FIGURE 2:**
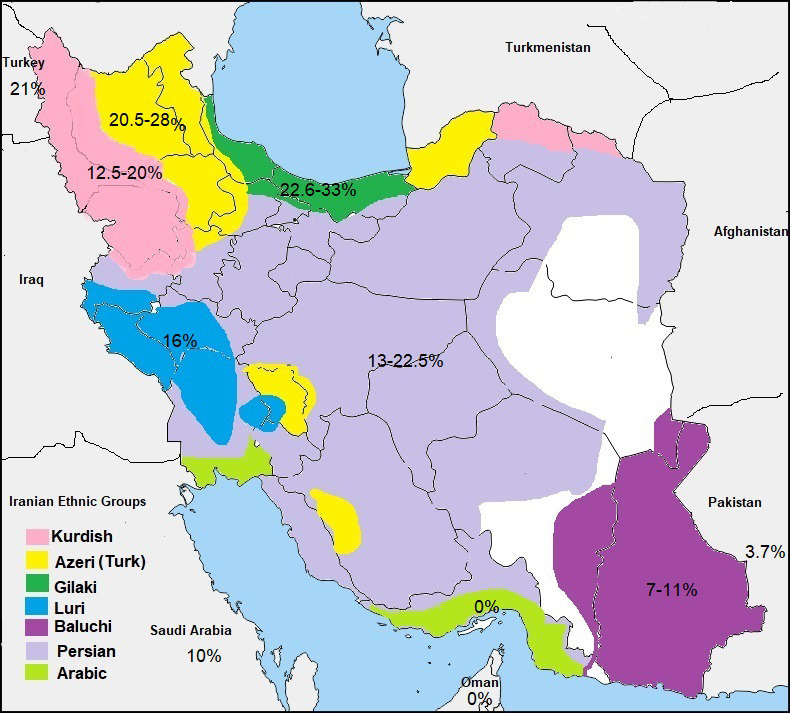
Prevalence of GJB2 mutations in various Iranian ethnic groups. The percent of 35delG mutation in Iran's neighbors (Turkey, Saudi Arabia, Oman, and Pakistan) and seven different Iranian ethnic groups, including Azeri,^11^, ^29^ Gilaki,^44^ Kurd,^11^, ^31^ Lur,^11^ Baluch,^28^ Fars,^11^, ^12^ and Arab^33^ are presented in the map

Hearing loss (HL) is the most common human‐inherited sensory abnormality that has become a worldwide public health concern. At least 1 in 500 newborns is affected by congenital hearing loss.^17^ Studies on various ethnic groups in several countries and nations have indicated that different genes associated with HL, especially mutations in the connexin 26 gene (GJB2), which is located in the DFNB1 locus (13q12), are the most common cause of ARNSHL.^18^ There are variations in the spectrum of GJB2 mutations in diverse societies, ranging from 87.7% in Finland,^19^ 57.5% in Lithuania,^20^ 45.6% in Slovakia,^21^ 33.3% in Croatia,^22^ and 25% in Turkey^23^ to 3.7% in Pakistan^24^ and 0% in Oman.^25^ Research on GJB2 mutations in Iran has indicated that the frequency of GJB2 mutations ranges between 0% and 35% across different ethnicities and regions of the country (Figure 2).^26^ This study as a continuation of a previous study conducted by Davarnia et al.^1^ on Iranian Azeri people with HL problems (Ardabil Province, the northwest of Iran) that showed a prevalence of 26% for GJB2‐related HL in Iranian Azeri people and reported 35delG as the most common HL‐causing variant. This result is comparable with those previously obtained by Najmabadi et al.^27^ who indicated that the highest percentage of GJB2‐related HL in Iran's north and northwest regions were 38.3% and 22.2%, respectively. They also demonstrated a gradual increase in the 35delG mutation frequency and GJB2‐related HL from the southeast to the northwest in the country, where people were connected to their neighbors in Pakistan and Turkey, respectively. In another research, Hashemzadeh Chaleshtori et al.^26^ reported a GJB2 mutation rate of 27.5% in the north and northwest of Iran and lower than 4% in the south and southeast of Iran. Naghavi et al.^28^ investigated GJB2 mutations in 100 families with ARNSHL in Sistan and Baluchistan Province in the southeast of Iran and detected GJB2 mutations in 7% of the families. Interestingly, they reported c.35delG; p. Gly12Val was absent in these families and c.71G>A c. Trp24X was the most common GJB2 mutation. This result is comparable with those from the Pakistani population. On the other hand, Bonyadi et al.^29^ in their study on Azeri Turkish patients with GJB2‐related deafness reported that GJB2 mutations were responsible for about 28% of Azeri patients with ARNSHL and that 35delG was the most common mutation, causing GJB2‐related HL.

**FIGURE 3:**
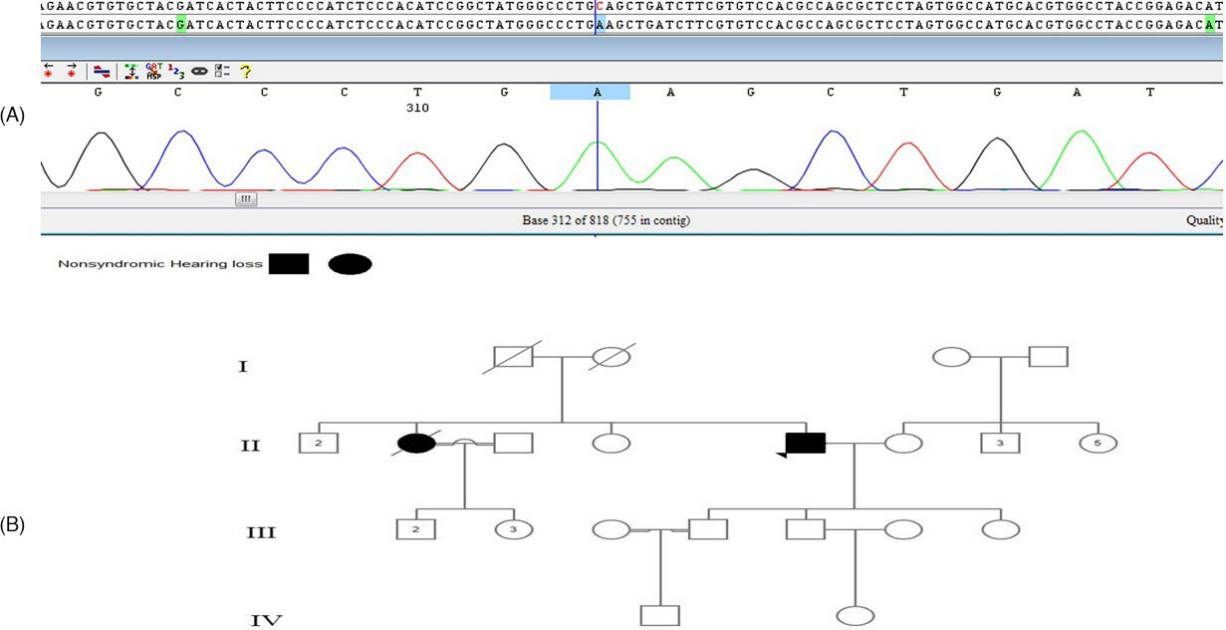
Sequences of the C to A transition (c.238 C>A) in the GJB2 gene of the proband (A); the pedigree of the family with the identified mutation (c.238 C>A) (B)

Families participating in this study lived in Ardabil in the northwest of Iran. According to previous findings, we expected a high prevalence of GJB2 mutations, leading to HL in this province. Our results confirm the critical role of GJB2 mutations in the Azeri ethnic group in the northwest of Iran. We indicated that GJB2 mutations caused 26% of ARNSHL cases in the Azeri population. We also observed 35delG as the most prevalent mutation, accounting for 61.5% of GJB2 mutations in the province. These findings are in agreement with those reported by Najmabadi et al., Davarnia et al., and Bonyadi et al. (Table 2).

**TABLE 2:** Type and frequency of GJB2 mutations in Ardabil Province in the past and present studies

Genotype	Effect on protein	Zygosity	Inheritance pattern	Consanguinity	Availability	Ref
35delG/35delG	p.Gly12Val	HOM	AR	6/9 Consanguineous	9/50	^1^
					16/81	^11^
					8/50	The present study
c.35delG/IVS1+1G>A	p.Gly12Val	HET		‐	1/81	^11^
c.551G>A/c.380G>A	p. R184Q p. R127H	HET		‐	1/81	^11^
358‐360delGAG/358‐360delGAG	p.delGlu120	HOM	AR	Consanguineous	1/50	^1^
					1/81	^11^
					2/50	The present study
463‐464delT/463‐464delT	p.Tyr155Met	HOM	AR	Consanguineous	2/50	^1^
					1/81	^11^
299‐300delAT/299‐300delAT	p. His100Arg	HOM	AR	Non‐consanguineous	1/50	^1^
					1/81	^11^
					1/50	The present study
c.238C>A/ c.238C>A (The first report in Iran)	p.Gln80Lys	HOM	AR	Non‐consanguineous	1/50	The present study
c.290dupA/c.290dupA	p.Tyr97Ter	HOM	AR	Non‐consanguineous	1/50	The present study
c.79G>A/314A>G	p.Val27Ile p.Glu114Gly	HET	AR	Consanguineous	1/50	^1^
c.511G>A/WT	p.Ala171Thr	HET	AR	Consanguineous	1/50	^11^

Since Iran's neighbor to the northwest is Turkey, we expected a comparable prevalence of different GJB2 mutations, resulting in ARNSHL, between the northwest of Iran and Turkey. A study performed by Kalay et al.^23^ in Turkey indicated that GJB2 mutations caused about 25% of ARNSHL cases in this country. In another study, Tekin et al.^30^ reported that GJB2 mutations caused approximately 22% of ARNSHL cases in Turkey.

In general, as the Iranian population comprises various ethnic groups living in different regions, the frequency of GJB2 mutations varies between and 35% among Iranian people, and the prevalence of each GJB2 mutation is different in each ethnic group.

According to our results, c.35delG; p.Gly12Val is the most frequent GJB2‐related mutation among the Azari population, which is in line with previous studies.^10^, ^11^ Similarly, several studies indicated that the c.35delG variant was the most common mutation in the GJB2 gene among Gilaki, Turkish (Azari), Kurdish, and Fars ethnicities, accounting for 58.4%–76.23%,^2^, ^11^ 64.5%–71.1%,^11^, ^29^ 58.6%–88.8%,^11^, ^31^ and 61.7%–71% of GJB2 mutations,^11^, ^12^ respectively. However, previous findings reported c.71G>A; p.Trp24X (75% of mutant alleles) as the most common GJB2‐related mutation among Sistani and Baluchi ethnic groups.^28^ This mutation has also been found to be the most detected variant in India.^32^ Surprisingly, Galehdari et al.^33^ in their study detected no GJB2‐related HL in families of Arabian origin.

The variation in GJB2 mutations among different ethnic groups may be due to human immigration, presenting a founder effect instead of a mutational hotspot. The high proportion of GJB2 mutations in the center and northwest of Iran might be due to immigration from other cities and countries, and most important consanguineous marriage among distinct ethnic groups. In addition, the evidence suggests a founder effect in the north of Iran (compared with 43% in Anatolia). Based on the prevalence of c.35delG; p.Gly12Val mutation among the Iranian population and due to the migration of tribes from central Asia to Anatolia about 10 centuries ago, we believe that c.35delG mutation originates in the northwest of Iran.^27^, ^34^ Also, we assume c.35delG mutation as a common mutation in Azerbaijan as people in this country is of a similar ethnicity as people in the northwest of Iran, which requires further studies.

In our study, the two homozygote c.358‐360delGAG; p. Glu120del mutations were identified (2/50). According to previous studies, in general, the first and second common GJB2 mutations in Iran^35^ and Turkey^30^ were c.35delG and c.358‐360delGAG, respectively, which is in line with our research results. The findings of a comprehensive study on the GJB2 mutations among Iranian population, which was conducted by N. Bazazzadegan et al., indicated that c.358–360delGAG; p. Glu120del variant manifested in a variety of causes in the patients of the GJB2‐related HL within Iranian population. This variant is more common among Azeri and Kurdish ethnicities (the second common GJB2 mutation) compared with Fars and Lur populations that are considered the third most prevalent mutation. Also, c.358‐360delGAG; p. Glu120del has a lower effect on GJB2‐related HL in Gilaki and Mazzani people. On the other hand, this study clarified that c.358‐360delGAG; p. Glu120del variant had no effect on the ARNSHL etiology in Arab and Baluchi population.^11^, ^28^, ^36^

Furthermore, the homozygote 299‐300delAT; p. His100Arg was found in one of the families in our study. Abe et al.^37^ identified this mutation as a heterozygote variant, leading to HL. Wang et al. discovered this variant in homozygote form in a patient,^38^ showing an autosomal recessive‐inherited GJB2 mutation related to HL. This mutation has also been identified in Turkey^39^ and Iran^11^ as a disease‐associated variant, leading to ARNSHL. Clearly, according to previous studies, 299‐300delAT; p. His100Arg mutation is prevalent among Iranian population,^11^ especially in the northwest of Iran among Azari ethnic.^1^, ^10^ In addition to Azeri population, this variant is reported in Fars, Lur, and Kurdish^11^, ^31^ ethnicities. Nevertheless, this mutation was not identified among Baluchi,^28^, ^40^ Arab,^40^, ^41^, ^42^ Gilaki, and Mazzani^2^, ^11^ population who live in the north and south of Iran.

Another important variant in this study was a homozygote c.290dupA; p. Tyr97Ter mutation. This variant was formerly reported by Bazazzadegan et al. and Bonyadi et al. among Fars and Azari ethnicities in Iran, respectively^10^, ^11^, ^29^ (Table 3). It is noticeable that c.290dupA; p. Tyr97Ter mutation has not been reported among Lur, Kurdish, Arab, Baluchi, and Gilaki population in Iran. Thus, we suggest that although c.290dupA; p. Tyr97Ter mutation has a significant effect on ARNSHL etiology among Fars and Azari population in Iran, it has no effect on other ethnicities.

**TABLE 3:** Percent of mutations in the present study in different Iranian ethnic groups compared with Bazazzadegan et al.’s study on the Iranian population

Variants	Azari (Turk)	Fars	Kurd	Arab	Baluchi	Lur	Gilaki and Mazzani	Our studies	Ref
c.35delG	76.1%	61.7%	58.69%	0	0	66.66%	76.23	61.5%	^11^
c.290DupA	0	0.5%	0	0	0	0	0	7.6%	
c.358‐360delGAG	5%	4.46%	15.2%	0	0	4.76%	1%	15.38%	
c.299‐300delAT	0.9%	1.24%	0	0	0	2.3%	0	7.6%	
c.238C>A (First report in Iran)	0	0	0	0	0	0	0	7.6%	

The novel variant identified in this study was c.238C>A; p. Gln80Lys that has not been reported in Iranian population so far. This mutation was identified as homozygous in a patient with severe HL (Figure 3). Kalay et al. first described c.238C>A; p. Gln80Lys in the Turkey population^23^ as a compound heterozygote variant related to HL with c.35delG in a patient with severe HL. The second transmembrane domain contains c.238C>A; p. Gln80Lys mutation that is conserved in all connexin families, indicating their critical function and structure. To date, four missense mutations (Q80K, Q80P, Q80R, and Q80H) and one nonsense mutation were reported at this position, which lead to HL and can be considered in the screening, identification, and study of people with deafness.^43^ Therefore, this novel mutation in the GJB2 gene appears to cause HL in the Iranian population, especially in the Azeri population. Therefore, the mutation screening of GJB2 for c.238C>A; p. Gln80Lys is recommended in patients with HL, belonging to the Azeri ethnic group.

Based on our findings, the high prevalence of c.35delG; p. Gly12Val compared with other damaging and pathogenic variants of the GJB2 gene in the Iranian population, especially Azeri people with ARNSHL, may be because of the founder effect, the high ratio of consanguineous marriages, and neighboring with Turkey, which has a high proportion of c.35delG in the GJB2 gene. Also, the lack of GJB6 mutations among the Iranian population may strengthen the founder's hypothesis. As a result, further research should be carried out to investigate other pathogenic variants in other loci. Also, the elevated penetrance of GJB2 mutations in the Azeri population may increase the possibility of genetic testing to detect newborns with ARNSHL, which can prevent inappropriate genetic tests and improve the diagnosis and treatment of ARNSHL in Iran.

## 5 CONCLUSION

No c.238C>A; p. Gln80Lys variant in the GJB2 gene has been detected in the Iranian population, which is in line with the results of this study and indicates that it is likely to have a significant effect on the ARNSHL etiology in Iranian population. Also, our findings support the idea that GJB2‐related HL has various profiles in different populations of Iran with c.35delG variant that is the most common mutation in GJB2‐related HL in the Iranian population, especially in the Azeri population, which is higher than the average.

## AUTHOR CONTRIBUTIONS

Ehsan Abbaspour, Mohammad Panahi, and Bahareh Rahimi contributed to sequence analysis, bioinformatics analysis, and patient data interpretation regarding sequencing. These authors also contributed to this work equally and together wrote the manuscript. Haleh Mokaber and Reza Farajollahi wrote some sections of the manuscript. Behzad Davarnia supervised, reviewed, and edited the manuscript. All the authors read and approved the final manuscript.

## Data Availability

Input data for the analyses are available from the corresponding authors on request.
